# Deceased Donor Kidney Transplantation Outcomes at a Sri Lankan Center: A Comprehensive Single-Center Analysis

**DOI:** 10.7759/cureus.39250

**Published:** 2023-05-19

**Authors:** Mohamed Mujahith Salahudeen Buhary Ahamed, Mohamed Nazar Abdul Latiff

**Affiliations:** 1 Nephrology, Postgraduate Institute of Medicine, Colombo, LKA; 2 Renal and Transplantation, Queen Alexandra Hospital, Portsmouth, GBR; 3 Nephrology, National Hospital of Sri Lanka, Colombo, LKA

**Keywords:** renal transplant one-year outcome, deceased donor kidney transplantation, renal transplant rejection, observational cross-sectional study, post-renal transplant complications

## Abstract

Background

Chronic kidney disease (CKD) causes significant morbidity and mortality in patients and incurs a huge burden on healthcare expenses globally. Renal replacement therapy becomes imperative when patients reach end-stage renal disease. Kidney transplant is the best modality of choice for the majority of patients, and deceased donor kidney transplantation is the major contributor in the majority of countries. We present an outcome study in Sri Lanka for deceased donor kidney transplantation.

Methodology

This is an observational study conducted at the Nephrology Unit 1 at the National Hospital of Sri Lanka, Colombo, in patients who had undergone deceased donor kidney transplantation from July 2018 to mid-2020. We studied the outcomes of these patients for one year, including delayed graft function, acute rejection, infection, and mortality. Ethical clearance was obtained from the ethical review committee of the National Hospital of Sri Lanka, Colombo, and the University of Colombo.

Results

The study included 27 participants with a mean age of 55 ± 9.519 years. Diabetes mellitus (69.2%), hypertension (11.5%), chronic glomerulonephritis (7.7%), chronic pyelonephritis (7.7%), and obstructive uropathy (3.8%) were the etiological factors of CKD. Basiliximab was used as an induction agent, and a tacrolimus-based triple-drug regimen was used for maintenance in all patients. The mean cold ischemic time was 9 ± 3.861 hours. The majority (44%) of recipients had an O-positive blood group. At one year, the mean serum creatinine was 1.40 ± 0.686 mg/dL, and the mean estimated glomerular filtration rate was 62 ± 21.281 mL/minute/1.73 m^2^. Delayed graft function occurred in 25.9% of the recipients, and 22.2% had acute transplant rejection. Postoperative infection was observed in 44.4% of recipients. One year after transplantation, 22% of the recipients died. Infection was the cause of death in 83% of recipients (five of six patients). The causes of death in the study sample were pneumonia (50%), including pneumocystis pneumonia (17%), myocardial infarction (17%), mucormycosis (16%), and other infections (17%). There was no significant association between outcomes at one year with age, gender, causes of CKD, or postoperative complications.

Conclusions

Our study found that the one-year survival rate following deceased donor kidney transplantation in Sri Lanka is relatively low, with infections being the leading cause of mortality. The high infection rate during the early post-transplant period underscores the need for enhanced infection prevention and control measures. Although we did not observe any significant association between the outcomes and the variables studied, it is important to note that the small sample size of our study may have influenced this finding. Future research with larger sample sizes may provide more insights into the factors influencing post-transplant outcomes in Sri Lanka.

## Introduction

Chronic kidney disease (CKD) is a growing problem worldwide, including in Sri Lanka, where CKD of uncertain origin threatens to reach epidemic proportions. Globally, the main contributors to CKD are diabetes and hypertension, which are also prevalent in Sri Lanka, along with various forms of glomerulonephritis. The increasing prevalence of non-communicable diseases, especially diabetes and hypertension, is expected to further burden the healthcare system of Sri Lanka [1,2].

End-stage renal disease (ESRD) is characterized by permanent kidney failure, requiring renal replacement therapies, such as dialysis or kidney transplantation, to sustain life. Kidney transplantation is the treatment of choice for most patients with ESRD as it improves the quality of life and reduces mortality risk compared with maintenance dialysis, and its superiority in prolonging the longevity of patients with ESRD is well established [3-5].

However, the biggest challenge in kidney transplantation is organ shortage, which makes the use of deceased donors increasingly important. In Sri Lanka, the number of living donor kidney transplantations has remained unchanged for years, while more than 2,000 patients died due to renal failure while awaiting transplantation in 2017 [6,7]. In contrast, approximately 34,768 organ transplants were performed in the United States in the same year. Studies on deceased donor kidney transplantation are scarce in Sri Lanka, although a recent surge in transplant operations following the implementation of the national transplant program has been observed at the National Hospital of Sri Lanka (NHSL), one of the few government hospitals with transplant facilities in Sri Lanka [8].

Limited research has been conducted on deceased donor kidney transplantation in Sri Lanka, with most studies focusing on living donor kidney transplantation. A study by Galabada et al., conducted at the Nephrology Unit 1, NHSL, found that the five-year death-censored graft survival rate for kidney transplant recipients from living donors was 93.5%, and the five-year patient survival rate was 82.2%, which is comparable to global transplant programs. This study identified the number of acute rejection episodes as an independent risk factor for graft survival [9]. A prospective study by Gunawansa et al. conducted over five years among live donor renal transplantations showed an overall patient survival rate of 86%, with a mean follow-up period of 36 months. Sepsis is the primary cause of death among recipients during follow-up, with graft pyelonephritis, pneumonia, and meningitis being the leading causes of sepsis [10]. In a retrospective analysis of renal transplant patients conducted by Rodrigo et al., the two-year survival rate of recipients was 75%, with a higher rate of post-transplant tuberculosis and fewer cytomegalovirus infections [11].

A study conducted by Gopalakrishnan et al. in India analyzed 173 cases of deceased donor renal transplants and identified the attainment of graft function immediately after transplant as the most crucial factor determining both graft and patient survival rates. The study reported an incidence of delayed graft function (DGF) of 48.5% and acute rejection of 21.8%. At one year, patient- and death-censored graft survival rates were 80% and 82.6%, respectively, while at five years, these rates were 76% and 80%, respectively [12]. Another retrospective study by Patel et al examined 236 deceased donor renal transplants performed from 2005 to 2011 and found patient and graft survival rates to be 77.08% and 86.8%, respectively. The study also observed a high one-year post-transplantation mortality rate, with sepsis being the leading cause of most deaths [13].

Since the establishment of the national deceased donor kidney transplant program in Sri Lanka, the number of deceased donor kidney transplants has increased significantly. The NHSL is among the few government hospitals with transplant facilities in Sri Lanka that has witnessed a recent surge in transplant operations after the launch of the national transplant program.

In the early transplant period, complications of renal transplantation can arbitrarily be thought to occur within the first three months of transplant surgery. During this period, several complications occur, including DGF caused by tubular necrosis, acute allograft dysfunction from various causes (e.g., acute rejection, ischemic tubular necrosis, nephrotoxic drugs, and infection), adverse side effects of drugs at higher doses (such as poor blood pressure control, drug toxicity, and poor glycemic control), surgery-related complications, and various infections that are more prevalent early in the transplant course.

This is the first study from Sri Lanka that focuses on deceased donor renal transplants. Our objective was to assess non-surgical complications that are specific to graft function and related to immunosuppressant medications. Specifically, we will examine DGF, acute allograft rejection, and infection, in addition to first-year mortality.

## Materials and methods

This study was a single-center, descriptive study of deceased donor kidney transplant recipients who underwent transplantation between July 2018 and September 2020 at the NHSL, Colombo. All patients who underwent deceased donor kidney transplants during the study period fulfilled the eligibility criteria and were included and followed up for one year. Ethical clearance for the study was obtained from the ethical review committees of the National Hospital of Sri Lanka, Colombo, and the University of Colombo (approval number: ETH/COM/2019/18, EC-19-016).

Patient demographic and clinical data were obtained from medical notes, clinical records, electronic records, and direct communication with patients. Written informed consent was obtained from all study participants. The baseline demographic characteristics were analyzed using descriptive statistics. Follow-up data were recorded on a Google spreadsheet, which included a preset checklist for monitoring complications during each patient review.

## Results

Our study included 27 patients who received deceased donor renal transplants between July 2018 and September 2020 and fulfilled the inclusion criteria. The mean age of the patients was 55 ± 9.519 years. Of the study sample, 22 (81%) were male and five (19%) were female, indicating a higher representation of males.

All patients received basiliximab-based induction agents and a tacrolimus-based triple-drug regimen for maintenance. After undergoing renal transplants, patients were managed in the surgical intensive care units and then moved to the vascular and transplant wards, where they were kept until discharge. However, in subsequent admissions for any reason, patients were admitted to general medical wards as we did not have a dedicated renal ward at that time.

Table 1 shows the clinical characteristics of the patients, including the etiology of CKD and mean cold ischemia time (CIT). Based on the collected data, diabetic nephropathy was the cause of CKD in 18 (69.2%) patients, while hypertension was the etiology in three (11.5%) cases. Chronic glomerulonephritis and chronic pyelonephritis were the causes in two (7.7%) patients each, while one (3.8%) patient had obstructive uropathy as the underlying cause.

**Table 1:** Clinical characteristics of the study sample.

Characteristics	Number (percentage %) (N = 27)
Diabetic nephropathy	18 (69.2)
Hypertension	3 (11.5)
Chronic glomerulonephritis	2 (7.7)
Chronic pyelonephritis	2 (7.7)
Obstructive uropathy	1 (3.8)
Cold ischemia time (hours)	9 ± 3.861

**Figure 1:**
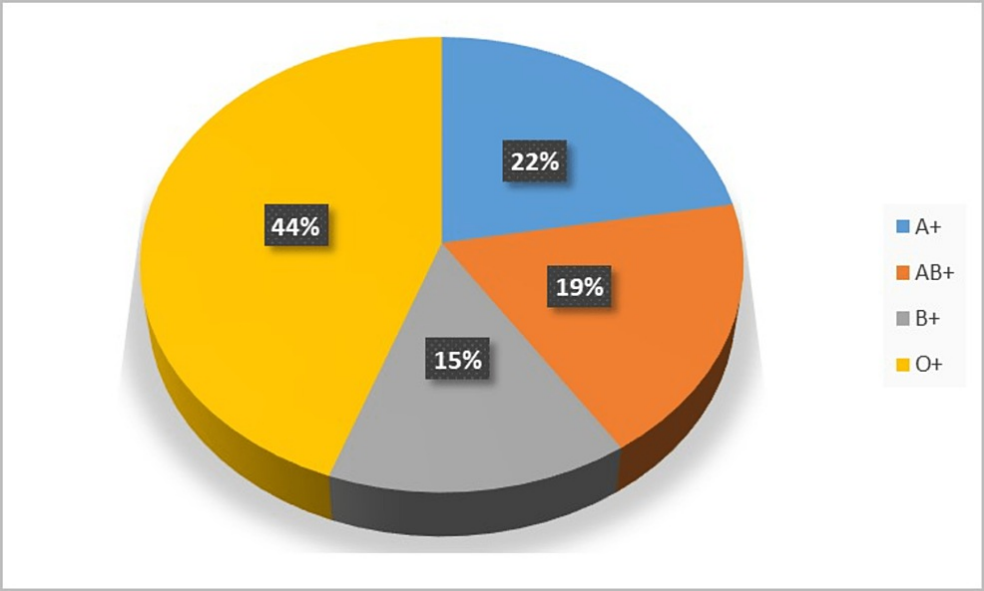
Blood group.

Figure 1 shows a pie chart of the distribution of blood groups among the patients who participated in the study. The chart shows that the majority of patients, 12 (44%), had blood group O+. Additionally, six (22%) patients had an A+ blood group, five (19%) had an AB+ blood group, and four (15%) had a B+ blood group.

**Figure 2:**
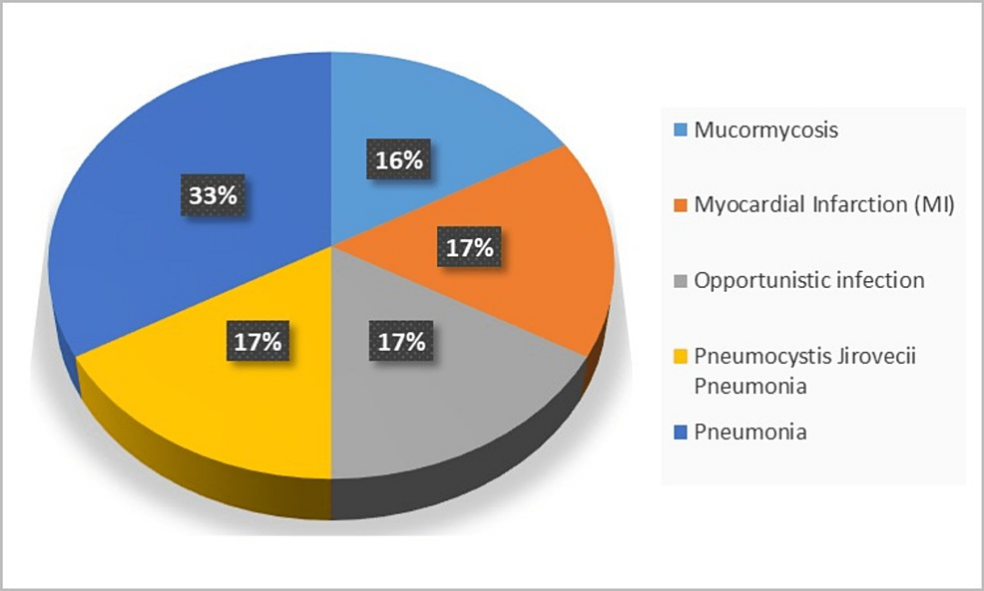
Cause of death.

The pie chart in Figure 2 illustrates the causes of death among the six patients who died. Three (50%) patients died due to pneumonia, including one (17%) with *Pneumocystis jirovecii* pneumonia. One (16%) patient died of mucormycosis, one (17%) patient died of myocardial infarction, and another patient (17%) died of another opportunistic infection. Notably, five of the six (83%) patients who died had infections as a contributing factor to their death.

**Figure 3:**
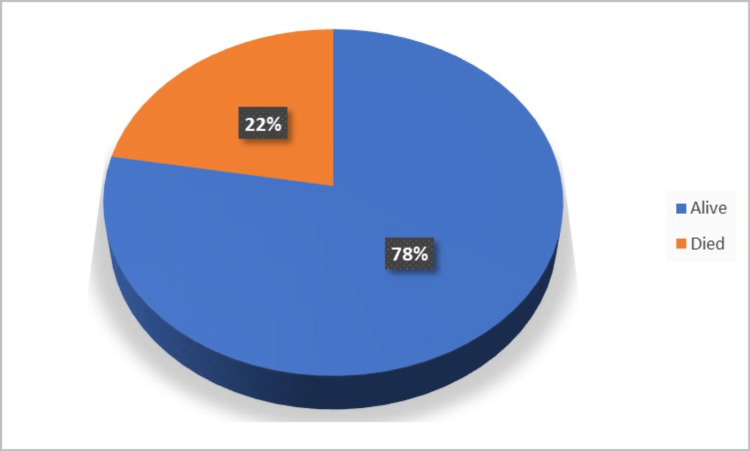
Outcome at one year.

The pie chart in Figure 3 illustrates the outcomes of deceased donor kidney transplants at the end of one year. Of the total recipients, six (22%) had passed away by the end of the first year, while the remaining 21 (78%) were still alive.

The data presented in Table 2 indicate that among the patients who were still alive at the end of the study period of one year, the mean serum creatinine was 1.40 ± 0.686 mg/dL and the mean estimated glomerular filtration rate (eGFR) was 62 ± 21.281 mL/minute.

**Table 2:** Serum creatinine and eGFR at one year. CKD-EPI equation was used to calculate the eGFR. CKD-EPI = Chronic Kidney Disease Epidemiology Collaboration; eGFR = estimated glomerular filtration rate

Parameter	Mean ± SD
Serum creatinine (mg/dL)	1.40 ± 0.686
eGFR (mL/minute)	62 ± 21.281

**Figure 4:**
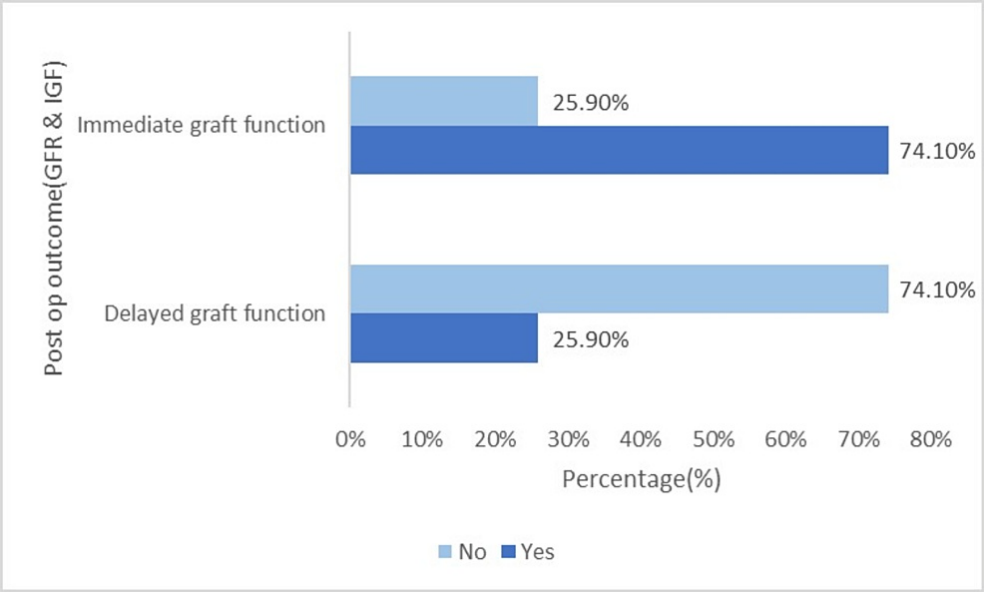
Postoperative outcome (DGF and IGF). DGF = delayed graft function; IGF = immediate graft function

Based on the horizontal bar chart in Figure 4, 20 (74.10%) patients experienced immediate postoperative graft function, indicating that they did not require dialysis following transplantation. Conversely, seven (25.90%) patients experienced DGF, requiring dialytic support within a week of transplantation.

**Figure 5:**
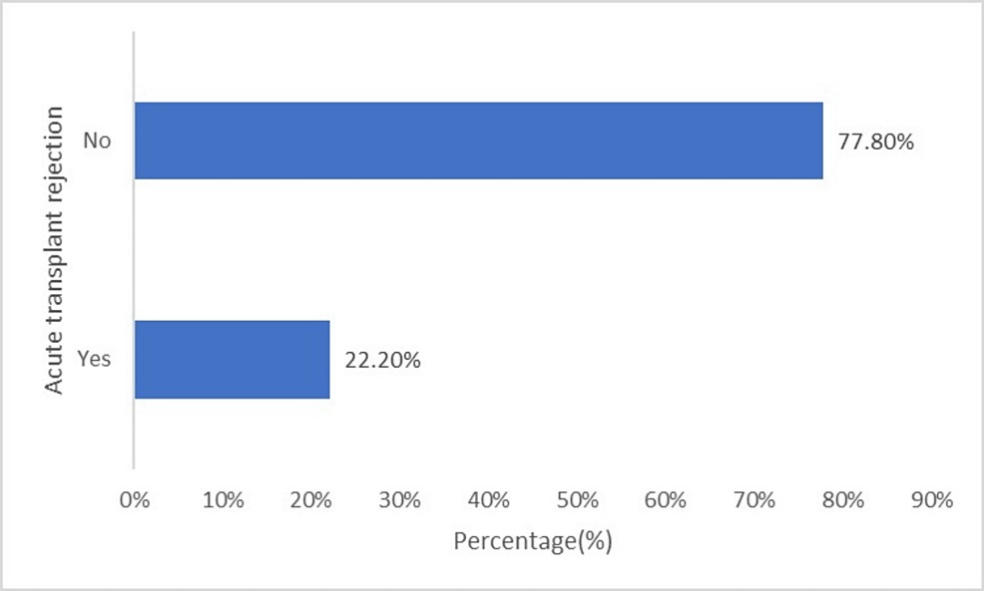
Acute transplant rejection.

The information presented in Figure 5 pertains to the incidence of acute transplant rejection in patients who underwent deceased donor kidney transplant surgery. The data show that out of the study sample, six (22.20%) patients experienced acute transplant rejection, while the remaining 21 (77.8%) patients did not exhibit any signs of acute rejection. Three patients had antibody-mediated rejection, two had acute T-cell-mediated rejection, and one had mixed rejection.

**Figure 6:**
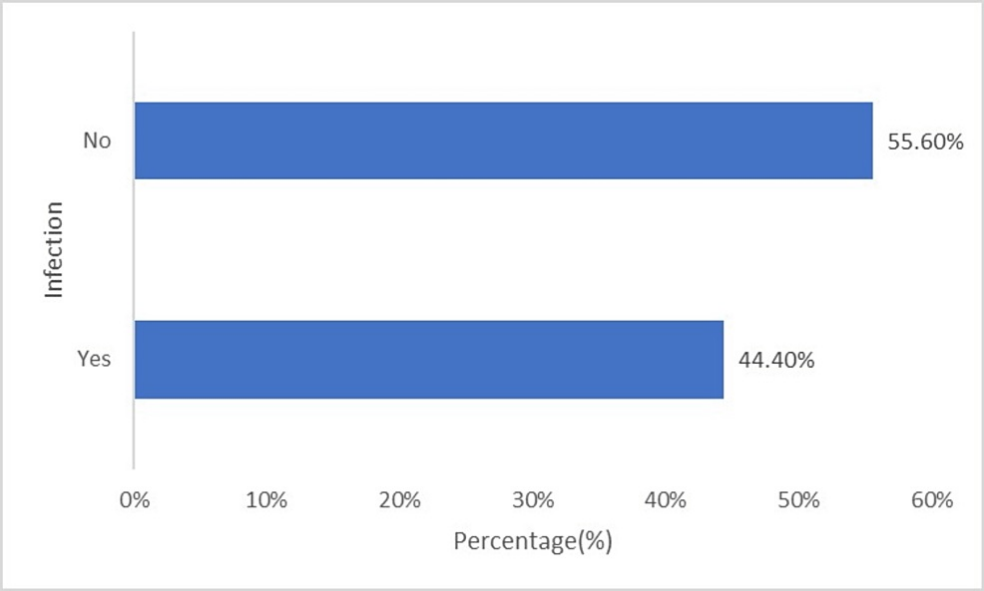
Post-transplantation infection.

The horizontal bar in Figure 6 shows that a very high proportion of recipients suffered from infection postoperatively. Twelve (44.4%) of the 27 patients studied experienced infection within one year of transplantation. The remaining 15 (55.6%) patients did not have any infections within the same period.

Table 3 presents the data analysis of the associations between the outcomes and demographic factors. The mean age of the patients who died following a deceased donor kidney transplant was 60 ± 5.565, and the mean age among the patients alive was 53 ± 10.002. All six (100%) patients who died were male. Sixteen (76.2%) males and five (23.8%) females were alive at one year. Among the study sample, five (100%) patients with diabetic nephropathy died, and the remaining 13 (61.9%) with diabetic nephropathy remained alive. In the blood group, among the dead, three (50.0%) were O+, two (33.3%) were A+, and one (16.7%) was AB+. None of these demographic factors had any significant association.

**Table 3:** Association between outcomes and demographic factors. *: Fisher’s exact value

Demographic factors	Outcome at one year		Significance
	Died (N = 6)	Alive (N = 21)	
Age (mean ± SD)	60 ± 5.565	53 ± 10.002	0.188
Gender			
Male	6 (100.0)	16 (76.2)	X^2^ = 1.75, df = 1, P = 0.555*
Female	0 (0.0)	5 (23.8)	
Cause of chronic kidney disease			
Diabetic nephropathy	5 (100.0)	13 (61.9)	X^2^ = 2.75, df = 1, P = 0.281*
Blood group			
A+	2 (33.3)	4 (19.0)	
AB+	0 (0.0)	5 (23.8)	
B+	1 (16.7)	3 (14.3)	
O+	3 (50.0)	9 (42.9)	

According to the data presented in Table 4 on the outcome at the end of one year in relation to the postoperative outcome, among the deceased patients, two (33.3%) had a complicated postoperative period with acute transplant rejection. Among the surviving patients, four ( 19.0%) had acute transplant rejection. Five (83.3%) patients whose postoperative period was complicated with infection died, and among the alive, seven (33.3%) were infected postoperatively, with a p-value of 0.060. DGF was observed in two (33.3%) of the patients who died, and five (23.8%) of the patients were alive by the end of one year.

**Table 4:** Postoperative outcomes and associations. *: Fisher’s exact value.

Postoperative outcome	Outcome at one year		Significance	OR (95% CI)
	Died (N = 6)	Alive (N = 21)		
Acute transplant rejection				
Yes	2 (33.3)	4 (19.0)	X^2^ = 0.551, df = 1, P = 0.588*	2.12 (0.283-15.96)
No	4 (66.7)	17 (81.0)		
Infection				
Yes	5 (83.3)	7(33.3)	X^2^ = 4.72, df = 1, P = 0.060*	10 (0.972-102.86)
No	1 (16.7)	14(66.7)		
Delayed graft function				
Yes	2 (33.3)	5 (23.8)	X^2^ = 0.220, df = 1, P = 0.633*	1.6 (0.223-11.49)
No	4 (66.7)	16 (76.2)		

**Figure 7:**
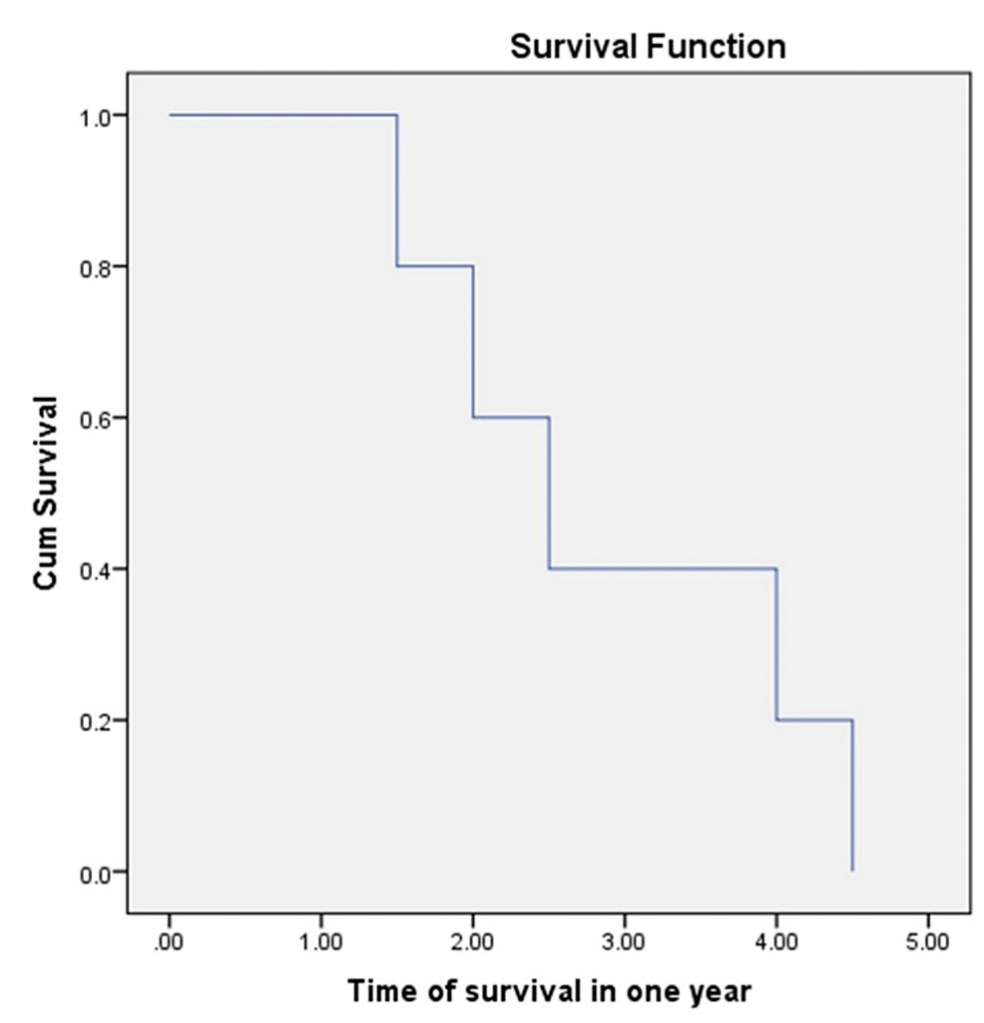
Time of survival in one year.

Figure 7 represents the time of survival in one year among the patients who died, where the data shows that in 2.5 months we lost 50% of our patients due to infection.

## Discussion

Owing to various etiological factors, CKD has become a significant health concern in Sri Lanka [14]. The worldwide prevalence of CKD was 13.4% in 2019 [15]. Renal replacement therapy is necessary to sustain life when the disease progresses to the end stage. Among these, kidney transplantation is considered superior to dialysis. Kidney transplantation improves the quality of life and reduces the healthcare burden of end-stage renal failure compared with dialysis [16]. However, kidney transplantation has shown several early and late complications, and 4.8% of post-transplantation patients were found to be returning to dialysis. Early recognition and prompt management of complications must be considered in graft [17].

Our study included all patients (n = 27) who underwent deceased donor kidney transplantation during the enrollment period. The mean age of the study sample was 55 ± 9.519 years, and there was a higher proportion of male patients.

The median CIT in our sample was 9 ± 3.861 hours. Many studies have revealed the importance of reducing CIT in the prevention of post-surgical complications. It was found that a CIT of more than 20 hours has a higher risk of developing DGF; however, there was no difference between patients with a CIT of more than 20 hours and less than 20 hours at the one-year follow-up [18,19]. The results of our study show that our study setting maintained a comparatively better CIT.

The blood group in the majority of the patients (44%) was O+, 22% were A+, 19% were AB+, and 15% were B+. ABO compatibility is an important factor in finding a kidney donor. However, recent studies have described that ABO-incompatible kidney transplantation can also result in better outcomes when the required amount of medical care is provided and can be used when compatible donors are not found [20,21].

When comparing our results to those of a previous study conducted by Awan et al. in the United States, we found that the leading cause of one-year mortality following kidney transplant was cardiovascular disease (24.7%), followed by infectious causes (15.2%). However, in a previous study, only 3.2% of patients died within one year of kidney transplantation. These differences in results can be attributed to variations in the sample size and follow-up duration between the two studies [22].

At one year of transplantation, the mean serum creatinine of the sample was 1.40 ± 0.68 mg/dL, and the mean eGFR was 62 ± 21.28 mL/minute/1.73 m^2^. Both of these investigations can be used as prognostic assessments in post-kidney transplantation patients. The median measured GFR, one year following kidney transplantation, was 55 mL/minute/1.73 m^2^, and eGFR values were found to be different from the measured GFR. These findings occur as a result of using different equations with different biochemical findings to calculate eGFR [23].

Following transplantation, 25.9% of our sample had DGF and required dialysis support. DGF is a salient factor in determining the survival of both patients and the graft and develops due to the progression of acute kidney injury in the grafted kidney. Anticipation of DGF during surgery and early recognition and management is recommended to prevent morbidity in organ recipients [24]. Acute transplant rejection occurred in 22.2% of the patients. Three had antibody-medicated rejection, two had acute T-cell-mediated rejection, and one had mixed rejection. DGF is a risk factor for acute graft rejection [25]. As demonstrated in previous studies, approximately similar percentages of our sample developed DGF and acute graft rejection. Immunosuppression and host factors contribute to the development of infections and determine morbidity and mortality following kidney transplantation [26].

The leading cause of death among post-kidney transplant patients in our study was infection (83%), particularly pneumonia (50% (n = 3) of total deaths; 17% (n = 1 ) had pneumocystis pneumonia). Other infectious causes of death included mucormycosis (16%) (n = 1) and other infections (17%). Myocardial infarction accounted for 17% (n = 1) of the total deaths, while one-year mortality was observed in 22% of our study sample.

No significant difference was found between demographic factors (i.e., age, sex), causes of CKD, and outcome (dead or alive) following kidney transplantation. There was no association between the development of postoperative complications, such as acute transplant rejection, infections, and DGF, and survival at one year. Data from our study demonstrated that within 2.5 months of kidney transplantation, 50% of the patients developed an infection. The blood group, DGF, CIT, and age failed to predict the survival of patients following kidney transplantation. However, some studies have demonstrated that DGF is a risk factor for mortality and reduces the frequency of one-year survival [27]. A Japanese study stated that acute graft rejection plays an important role in determining the long-term survival of the graft [28].

Deceased donor kidney transplantation is an efficacious treatment option for end-stage renal failure, which results in long-term survival and an enhanced quality of life. Predicting complications due to kidney transplantation is important for the survival of patients and for reducing the healthcare burden of the country.

Our study had several limitations that must be considered when interpreting the findings. One major limitation was the high incidence of infections post-transplant, which was likely because most post-transplant patients were admitted to general medical wards after the initial postoperative period, for any indication, as our center lacked dedicated renal wards. This lack of specialized care likely contributed to the higher rates of infections and mortality, leading to relatively low one-year survival rates in our study population.

Additionally, our study had a relatively small sample size as deceased donor kidney transplantation was only recently initiated at our center. A larger sample size with a better probability and sampling technique would improve the generalizability of our findings and allow for a more robust investigation of deceased donor kidney transplantation outcomes in our population.

Another limitation of our study is the potential for interviewer bias as the data were collected using an interviewer-based method. Future studies should employ more objective data collection methods to mitigate this limitation.

Overall, despite these limitations, our study provides valuable insights into the outcomes of deceased donor kidney transplantation in our center and highlights the need for dedicated renal wards and enhanced infection prevention measures to improve post-transplant outcomes in our population.

## Conclusions

Kidney transplant is a lifesaving treatment modality for end-stage renal failure. However, complications due to surgery, immunosuppression, and host factors determine morbidity and mortality following kidney transplantation. Maintaining a lower CIT, taking adequate precautions to minimize the risk of infections, and minimizing the occurrence of DGF may improve patient and graft survival.
